# Femoral Vessel Occlusion Enhances Cardiac and Cerebral Perfusion in a Porcine Model of Cardiac Arrest

**DOI:** 10.1161/JAHA.124.037413

**Published:** 2025-06-23

**Authors:** Joshua Y. Kim, Benjamin Usry, Maren L. Downing, Samuel W. Seigler, Heather Holman, Jennie H. Kwon, Kristi Helke, Rupak Mukherjee, Jeffrey A. Jones, Kristen M. Quinn

**Affiliations:** ^1^ College of Medicine Medical University of South Carolina Charleston SC USA; ^2^ School of Osteopathic Medicine Campbell University Lillington NC USA; ^3^ Division of Cardiothoracic Surgery Medical University of South Carolina Charleston SC USA; ^4^ Department of Surgery Medical University of South Carolina Charleston SC USA; ^5^ Department of Comparative Medicine Medical University of South Carolina Charleston SC USA; ^6^ Research Service Ralph H. Johnson Veterans Affairs Health Care System Charleston SC USA

**Keywords:** cardiac arrest, cardiac perfusion, cerebral perfusion, CPR adjuncts, vascular occlusion, Coronary Circulation

## Abstract

**Background:**

Closed chest compressions during cardiopulmonary resuscitation (CPR) mechanically circulate blood to the organs during cardiac arrest, yet cardiac arrest remains among the most fatal diseases, with a mortality rate that exceeds 85% to 90% globally. Novel methodologies to improve organ perfusion, particularly in resource‐restricted settings, are overdue. This study evaluated the efficacy of external femoral vessel occlusion (FVO) during CPR in a large mammal model.

**Methods:**

Thirteen adult Yorkshire pigs were instrumented with vascular and electrophysiologic monitoring lines. Hemodynamic measures and cardiac and cerebral perfusion in the pre‐ and postarrest conditions were quantified via fluorescent microspheres infused into the circulation. Control (n=7) animals underwent routine CPR, whereas experimental (n=6) animals received CPR and FVO via external compression to the femoral vessels during the entirety of the 30‐minute resuscitative phase. The primary outcome was mean arterial pressure, and secondary outcomes included cerebral and cardiac perfusion.

**Results:**

During native heart function, external FVO demonstrated a significant increase in mean arterial pressure (73±3 versus 62±2 mm Hg, *P*<0.001). During cardiac arrest, animals undergoing CPR with FVO had a significantly higher mean arterial pressure compared with CPR alone (49±9 versus 32±3 mm Hg, *P*<0.001). CPR with FVO significantly increased cardiac (181 versus 80 mean fluorescence intensity, *P*=0.014) and cerebral perfusion (119 versus 27 mean fluorescence intensity, *P*<0.001).

**Conclusions:**

CPR with FVO significantly increased mean arterial pressure, cardiac perfusion, and cerebral perfusion over CPR alone. These findings suggest FVO may represent a novel adjunctive strategy and therapeutic opportunity to enhance cerebral and cardiac perfusion, thereby decreasing cardiac arrest morbidity and mortality.

**Nonstandard Abbreviations and Acronyms**
CA: cardiac arrest
CPP: coronary perfusion pressure
FVO: femoral vessel occlusion
MFI: mean fluorescence intensity
REBOA: resuscitative endovascular balloon occlusion of the aorta
ROSC: return of spontaneous circulation

**Research Perspective**

**What New Question Does This Study Raise?**

- Current cardiac arrest care has plateaued in effectiveness.
- External femoral vessel occlusion bilaterally with ongoing cardiopulmonary resuscitation raised mean arterial pressure and increased cardiac and cerebral perfusion significantly greater than cardiopulmonary resuscitation alone in a large mammal model of cardiac arrest.

**What Question Should Be Addressed Next?**

- Novel adjunctive therapies, such as femoral vessel occlusion, must be studied for their potential role in combating the historically poor outcomes from cardiac arrest.

Cardiac arrest (CA) remains one of the most lethal public health problems in the world. Closed chest compressions are the mainstay of cardiopulmonary resuscitation (CPR) and the standard of care treatment for CA since the 1960s.^1^ Despite widespread CPR training and implementation, cardiac arrest remains the largest cause of natural death in the United States.^2^ In 2020 in the United States, only 9% of adults survived to hospital discharge following an out‐of‐hospital cardiac arrest, and just 23.3% survived to hospital discharge following an in‐hospital cardiac arrest.^3^

Patient survival following CA is largely dependent on cardiac and cerebral perfusion, and neurologic outcomes are critically impacted by cerebral flow during the event.^4^ Chest compressions alone during CPR restore only 10% to 30% of normal coronary or cerebral perfusion.^4^ Pharmacologic therapies, like the injection of epinephrine, are used to increase arterial blood pressure during CA; however, benefit for long‐term survival in humans remains a topic of debate.^5^, ^6^, ^7^

The therapeutic benefits of current treatments for CA seem to have plateaued, and with continued poor outcomes, novel adjunctive strategies are emerging. The intra‐aortic resuscitative endovascular balloon occlusion of the aorta (REBOA) was originally developed for hemorrhage control; use of the REBOA decreases the need for blood products and results in higher survival rates in patients with blunt or penetrating trauma.^8^, ^9^ More recently the REBOA has shown promise as a CPR device.^5^ The use of the REBOA during CPR in animals experiencing CA significantly increased blood pressure following occlusion of the distal aorta.^10^, ^11^, ^12^, ^13^ Furthermore, a small case series demonstrated the REBOA's ability to increase systolic blood pressure and coronary artery perfusion pressure in humans undergoing cardiac massage for CA.^14^ These exciting results have prompted further research, such as the ongoing REBOARREST trial (Resuscitative Endovascular Balloon Occlusion of the Aorta in Non‐traumatic Out of Hospital Cardiac Arrest), to extend its use in the population of patients with CA.^15^ However, the invasiveness of its design and the requirement of sterile deployment by physicians make the REBOA difficult to adopt for CA events that occur outside of hospitals (out‐of‐hospital cardiac arrest).

External lower extremity compression is noninvasive and may be used for a wide range of clinical indications, including chronic venous insufficiency, orthostatic or postural hypotension, and to mitigate bleeding from trauma or percutaneous access. External compression has been shown to increase stroke volume and cardiac output.^16^ We hypothesized that proximal arterial and venous occlusion, described hereafter as femoral vessel occlusion (FVO), would increase mean arterial blood pressure (MAP) during CA, resulting in greater cardiac and cerebral perfusion.

Investigating the effectiveness of CPR adjuncts in humans is challenging due to patient comorbidities, underlying cause, ethical considerations, and the emergent and unpredictable nature of CA.^17^, ^18^ The porcine model has been adopted to study CA due to the animal's large size, ability to tolerate invasive procedures, similar baseline hemodynamics to humans, high blood volume, extensively studied neuroanatomy, and large chest that allows for sufficient force to be used during chest compressions.^19^ Here we describe a porcine model of CA to test the hypothesis that FVO increases MAP and cerebral and cardiac perfusion during CPR.

## METHODS

The data that support the findings of this study are available from the corresponding author upon reasonable request.

### Animal Preparation

The animal protocol was reviewed and approved by the institution's animal care and use committee. Thirteen domestic Yorkshire farm pigs were acclimated in the animal facilities for a minimum of 2 days before experimentation. There were 5 males and 11 females, aged between 2 and 3 months, weighing 31 to 50.6 kg. The animals were examined by veterinarians and were fasted for 24 hours before surgery, except for free access to water. The pigs were randomized into either the control or experimental group using a random number generator. On the day of experimentation, the animals underwent general anesthesia induction with ketamine (22 mg/kg), acepromazine (1.1 mg/kg), and atropine (0.04 mg/kg), and were intubated with a cuffed endotracheal tube and ventilated at a flow rate of 22 mL/kg per minute. The animals were positioned supine in the operating suite atop the Life‐Stat CPR device baseboard (Michigan Instruments). Pulse and oxygen saturation were measured continuously via pulse oximetry on the ear and monitored continuously by veterinary staff to ensure a complete and stable plane of general anesthesia.

### Cannulation and Isolation of Vasculature

The animals were positioned for surgical dissection of the cervical and femoral vasculature and subsequent cannulation. The carotid artery was dissected and cannulated with a 4 French catheter for arterial blood sampling. A 4 French catheter was also inserted into the right subclavian artery, and a 5 French catheter cannulated the internal jugular vein. All transducing catheters were secured in place with silk sutures. Hemodynamic pressures were measured via traditional arterial line in the carotid, externally secured. In the groin, bilateral 4 cm oblique incisions were made at the midpoint between the anterior superior iliac spine and pubis. The incisions were deepened to visualize the inguinal ligaments, which were retracted superiorly to expose the femoral vasculature. The femoral artery and vein were gently isolated and encircled with individual tagged vessel loops.

### Phase 1: Hemodynamics During Native Heart Function

**Figure 1:**
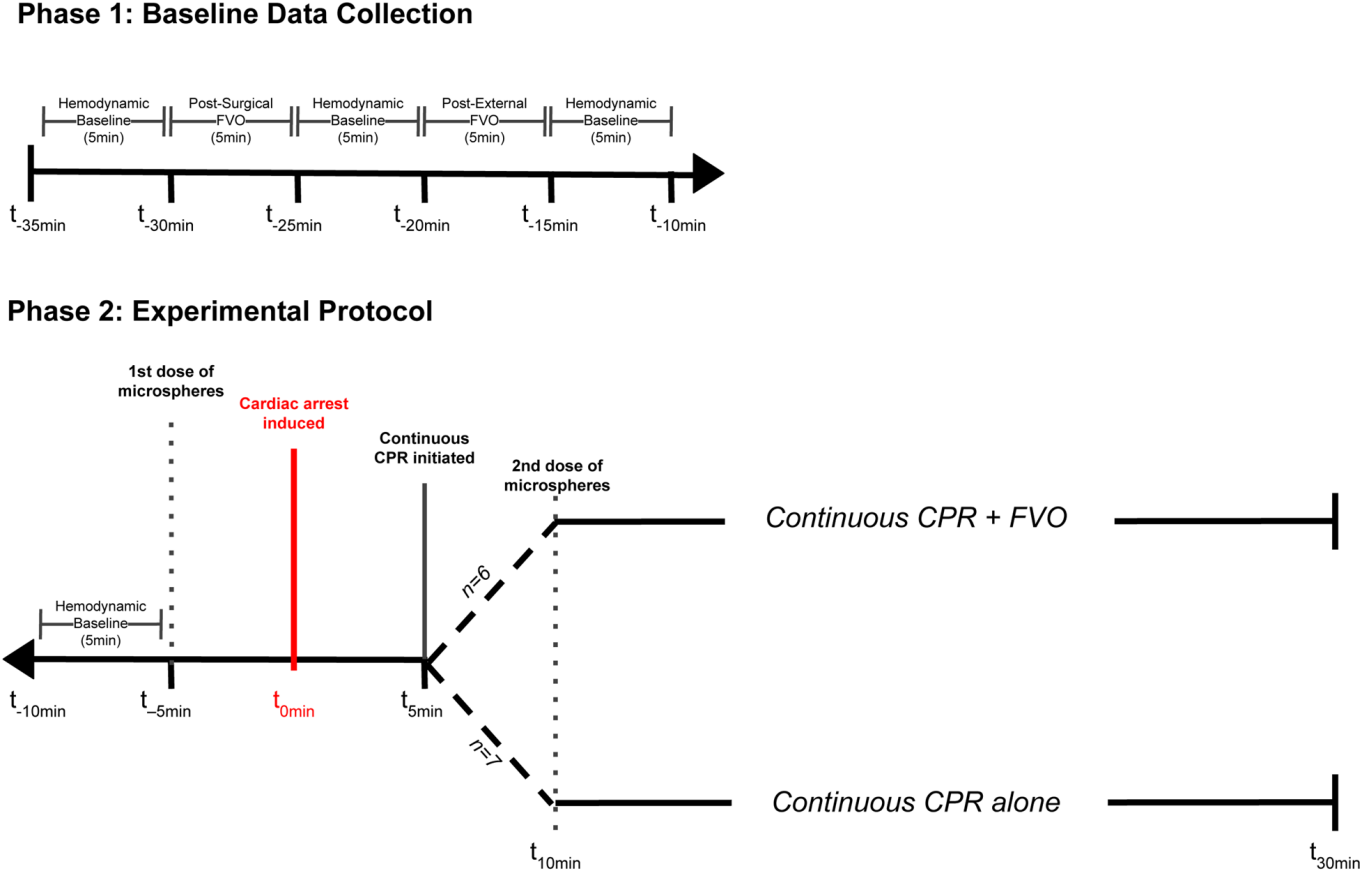
Experimental timeline. Phase 1 denotes the comparison of surgical FVO with external FVO during native cardiac function. Phase 2 denotes the administration times of the fluorescently tagged microspheres, induction of cardiac arrest (time 0), time that resuscitative (CPR) measures began, and when the second microsphere time point occurred. CPR indicates cardiopulmonary resuscitation; and FVO, femoral vessel occlusion.

Baseline hemodynamic values of carotid systolic and diastolic blood pressure, heart rate, temperature, end‐tidal CO_2_, and oxygen saturation were recorded at 1‐minute intervals from the arterial line and the anesthesia monitoring machine. After 5 minutes of physiologic baseline data collection, surgical FVO was performed for 5 minutes, defined as vessel loop occlusion of the isolated femoral artery and vein bilaterally. This period was followed by 5 minutes of a return to baseline physiological function. The same time intervals were compared with external FVO, defined as constant groin pressure applied manually over the femoral vessels by research staff for 5 minutes. The surgical FVO and external FVO conditions were randomized to occur as first or second in the experimental timeline for each animal (n=13). The timeline of this experimental phase is outlined in Figure 1.

### Phase 2: Hemodynamics During Cardiac Arrest

To prepare for microsphere infusion to measure perfusion, cannulation of the left ventricle was required. An incision was made at the level of the fourth intercostal space, and the left lung was retracted. A 5 French catheter was inserted into the left atrium through a small atriotomy, advanced into the left ventricle, and secured in place with a purse‐string suture. Following thoracotomy and cannulation of the left heart, we reapproximated the pericardium to prevent cardiac herniation to preserve chest wall mechanics. The Life‐Stat CPR device was fixed to the baseplate beneath the animal in preparation for CPR. The device's massager pad surface at the end of the piston was positioned directly over the left ventricle.

Microspheres (15 μm diameter, ThermoFisher Scientific FluoSpheres Polystyrene) were used to quantify heart and brain perfusion.^20^ Microsphere vials were protected from light and dispersed in solution using a vortex mixer and a sonicating water bath. The homogenized solution containing microspheres (6 mL) was injected by cannula into the chamber of the left ventricle over 30 seconds, followed by a 20 mL flush of heparinized saline over 30 seconds. While the heparinized saline was being flushed into the cannula, 14 mL of blood were withdrawn from the subclavian arterial line over 2 minutes. Five minutes before the induction of CA, baseline arterial blood gas was drawn, and microspheres were infused to quantify perfusion during native heart function.

CA was induced via electrocautery at 30 W applied directly to the epicardium of the heart, inducing ventricular fibrillation, as previously described.^19^ Ventilation was held at the time of arrest. No intervention was performed for 5 minutes, as previously described.^19^ Standardized chest compressions were initiated at 5 minutes post‐CA using the calibrated Life‐Stat Device at a rate of 100 compressions per minute at a minimum depth of 5 cm per the American Heart Association recommendation for CPR in adult humans.^20^ Animals were randomly selected to receive CPR+FVO (n=6) or serve as a control, CPR only (n=7). At 5 minutes after the initiation of CPR (10 minutes after induction of CA), a second arterial blood gas was drawn, and microspheres with a spectrofluorimetrically distinct color from the first administration were infused to determine perfusion during CA with resuscitation efforts.^21^ In the CPR+FVO condition, external FVO was performed by a study team member, not unlike the clinical role of holding pressure to a femoral access site to ensure hemostasis.

After 30 minutes of CPR, compressions were stopped, and the experiment was concluded. The full timeline is depicted in Figure 1. No vasoactive medications or defibrillation was performed, and no animals were kept alive. Tissue samples from the myocardium, cerebral tissue, and sartorius leg muscle were collected. The sartorius muscle samples were processed for histology (hematoxylin and eosin stained) to assess ischemic changes between control and experimental pigs by an expert veterinary pathologist.

### Microsphere Processing

Myocardial and brain samples were collected with assistance from the institution's veterinarian and veterinary staff. Samples (3 g each) were collected from 11 anatomic locations in the heart and 10 locations from the brain. Myocardial samples were taken from the left ventricular apex, proximal anterior septum, basal right ventricle, epicardium, endocardium, right ventricular lateral wall, left ventricular lateral wall, mitral papillary muscle, 2 from the anterior left ventricle, and the distal septum. The brain samples were taken from the bilateral dorsomedial cortex, ventromedial cortex, and midbrain. Biologic samples were digested in 30 mL of 10% potassium hydroxide and vortexed at 24‐hour intervals for a total of 48 hours at 37 °C. After completely digesting, the samples were centrifuged at ~1200*g* for 20 minutes. The supernatant was disposed of, and the pellets were resuspended in 5 mL of 0.5% Tween‐80. After washing, these samples were centrifuged again for 20 minutes. The supernatant was disposed of, and the pellets were then placed in 3 mL of 2‐ethoxyethyl acetate for 5 days in the dark at room temperature. After 5 days, the samples were spun down a final time, and the supernatant was transferred into individual wells on a 96‐well plate for spectrofluorimetric analysis. Pre‐CA fluorescence was measured with yellow wavelengths (515/534 nm). Post‐CA fluorescence was measured with red‐orange wavelengths (565/580 nm). Sample measurements were averaged from all sites in either the heart or brain.

### Statistical Analysis

Sample size required for this study was determined by performing an a priori power analysis using data from a past study conducted by Dogan et al.^22^ The power analysis determined a recommended sample size of 6 pigs per group (Figure S1). The primary outcome of this study, the MAP, was calculated from real‐time systolic and diastolic pressures recorded by the arterial transducer connected directly to the cannula in the carotid artery of each pig. These values were measured every minute during each phase of the experiment. The average, without exclusion, of these values was used for statistical analysis. Due to the smaller sample size, a Kruskal‐Wallis test was performed for the arterial blood gas measurements, and 3 independent Mann‐Whitney *U* tests were performed for mean separation between groups (Tables S1 and S2). Because the groups differed during baseline measure, we calculated a ∆MAP, defined as the difference of each specific animal's minute‐by‐minute MAP during CPR from its baseline pre‐CPR MAP (Table S3). This allowed each pig to serve as its own control and isolate the experimental differences. Secondary end points included the arterial blood gas measurements, serum chemistries, and mean fluorescence intensity (MFI). MFI is defined as the average fluorescence measured by spectrofluorimetric analysis of the organ samples from the animals' hearts and brains. The Student *t* test and ANOVA analysis were used to determine the separation of means between variables. Values are presented as mean±SD. *P* values <0.05 were considered statistically significant.

## RESULTS

Pigs of both sexes (males, n=4; females, n=9) were equivalently distributed between the groups. Seven pigs (2 males and 5 females) were assigned to the control group to receive standard CPR, and 6 pigs (2 males and 4 females) were assigned to the experimental group to receive CPR+FVO. The mean weight of the control group was 45.3±5.10 kg, and the mean weight in the experimental group was 40.2±5.46 kg (*P*=0.11). Baseline clinical data and comparison of the surgical versus external FVO during native heart function are summarized in Table 1. There was a statistically significant increase in MAP with external FVO compared with the control (*P*<0.001) and compared with surgical FVO (*P*<0.01). No other metrics differed between conditions, including end‐tidal CO_2_, heart rate, oxygen saturation, or temperature.

**Table 1:** Clinical Parameters During Native Heart Function (n=13)

	Baseline	Surgical FVO	External FVO	*P* value
Carotid MAP, mm Hg	62±2	67±2	73±3	<0.001
End‐tidal CO_2_, mm Hg	41±1	39±1	39±1	0.973
Heart rate, bpm	103±1	106±1	104±2	0.648
O_2_ saturation, %	98±1	98±1	97±1	0.517
Temperature, F	95.3±0.2	94.9±0.2	95.0±0.2	0.938

Hemodynamic and clinical data points during native heart function include comparison between surgical FVO with vessel loops and external FVO with occlusive device. N=13 for all conditions. All data points are presented as mean±SD. *P* values were calculated using a *t* test to compare between surgical femoral vessel occlusion and external femoral vessel occlusion. Baseline values are displayed to provide context to the hemodynamic parameters during native heart function. FVO indicates femoral vessel occlusion; and MAP, mean arterial pressure.

**Figure 2:**
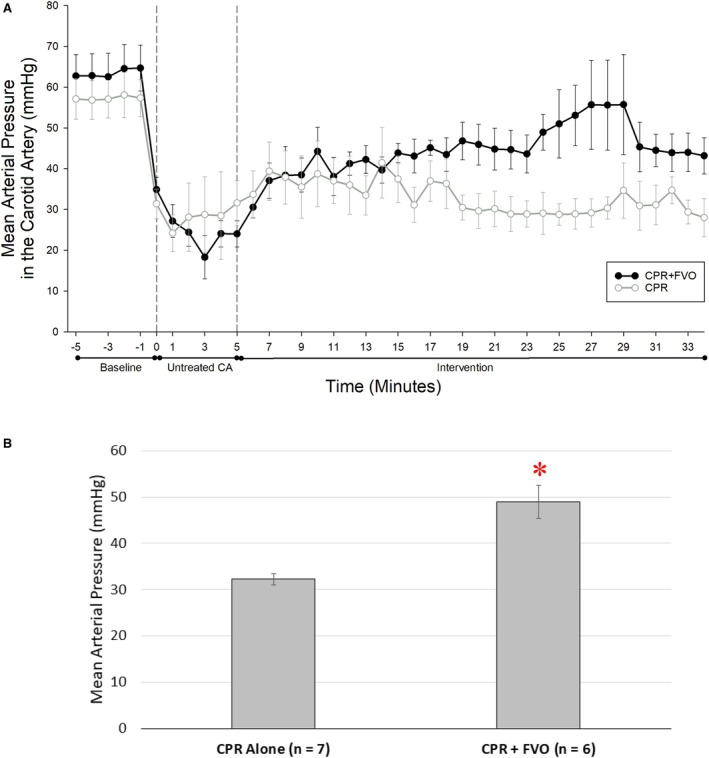
MAP during cardiac arrest. MAP during CA during CPR alone (n=7) and CPR+FVO (n=6) conditions. **A**, MAP recorded at each minute during CA and CPR is plotted over time, starting with baseline recordings; time 0 represents induction of CA followed by 5 minutes of untreated cardiac arrest, and then the respective intervention arms. Values are the mean from all animals within each group at that time point. **B**, The average MAP over the entire CPR period was 32±3 mm Hg in the CPR‐only group and 49±9 mm Hg in the CPR+FVO group (*P<0.05). CA indicates cardiac arrest; CPR, cardiopulmonary resuscitation; FVO, femoral vessel occlusion; and MAP, mean arterial pressure.

Clinical parameters recorded during pre‐CA baseline and then during CA between the experimental (CPR+FVO) and control (CPR) groups are summarized in Table 2 and Figure 2. Because this was a terminal experiment, no efforts for warming were undertaken during the procedures. Table S3 and Figure S2 detail ∆MAP, comparing the changes from respective baseline (pre‐CA) MAP between CPR+FVO pigs to CPR pigs. The ∆MAP was statistically significant between the experimental and control groups, with the CPR+FVO pigs having an average reduction in MAP of 18.5 mm Hg and the CPR‐only pigs having an average reduction of 22.6 mm Hg (*P*=0.0201). Additionally, arterial blood gas measurements were drawn 5 minutes before the induction of CA and 5 minutes after CPR or CPR+FVO had begun. Table 3 demonstrates that there were no significant differences in the biochemical values when comparing the CPR versus the CPR+FVO groups using a Student *t* test at 5 minutes of resuscitation. ANOVA analysis comparing these conditions with the pre‐CA condition found statistically significant differences in partial pressure of O_2_, bicarbonate, and end‐tidal CO_2_ (Table S4).

**Table 2:** Clinical Data Points During Resuscitation With CPR+FVO Versus CPR Alone

	Baseline (n=13)	CA (n=13)	CPR+FVO (n=6)	CPR (n=7)	*P* value
Carotid MAP, mm Hg	68±1.5	26.7±6.7	49±9	32±3	<0.001
End‐tidal CO_2_, mm Hg	38±0.8	19±8.4	18±5	14±6	<0.001
O_2_ saturation, %	98±0.16	49±43	63±20	71±66	0.174
Temperature, F	94±0.14	93±0.14	93±0.2	93±0.1	0.178

Hemodynamic and clinical data points during resuscitation with CPR+FVO vs CPR alone. Values are presented as mean±SD. *P* values were calculated using a *t* test or Kruskal‐Wallis test to compare between CPR+FVO and CPR groups. Baseline is included to provide context to hemodynamic parameters during resuscitation. Baseline values were taken from the 5 minutes before induction of CA. CA indicates cardiac arrest; CPR, cardiopulmonary resuscitation; FVO, femoral vessel occlusion; and MAP, mean arterial pressure.

**Table 3:** Arterial Blood Gas Measurements After 5 Minutes of Resuscitation With Either CPR+FVO Versus CPR Alone

Arterial blood gas values	CPR+FVO	CPR	*P* value
pH	7.32±0.19	7.35±0.32	0.748
pCO_2_	46.68±24.79	44.88±34.36	0.821
pO_2_	173.25±250.84	217.20±140.27	0.499
BEecf	−4.50±11.64	−3.20±10.87	0.710
Bicarbonate	21.73±10.67	22.06±8.05	0.703
TCO_2_	23.25±10.87	23.40±8.68	0.738
SO_2_%	73.75±33.00	89.60±22.14	0.764
Sodium	144.50±7.53	142.60±6.43	0.935
Potassium	3.80±1.00	3.80±1.07	0.928
iCA	1.04±0.31	1.16±0.31	0.871
Glucose ±	92.50±50.91	83.60±26.92	0.936
Hematocrit	21.67±7.14	21.00±5.36	0.877
Hemoglobin	7.37±2.55	5.85±4.08	0.518

N=9 (CPR+FVO, n=4; CPR, n=5). Values are presented as mean±SD. *P* values were calculated using a *t* test to compare CPR+FVO and CPR. BEecf indicates extracellular base excess; CPR, cardiopulmonary resuscitation; FVO, femoral vessel occlusion; iCA, ionized calcium; pCO_2_, partial pressure of carbon dioxide; pH, potential of hydrogen; pO_2_, partial pressure of O_2_; SO_2_%, oxygen saturation; and TCO_2_, total carbon dioxide content.

**Figure 3:**
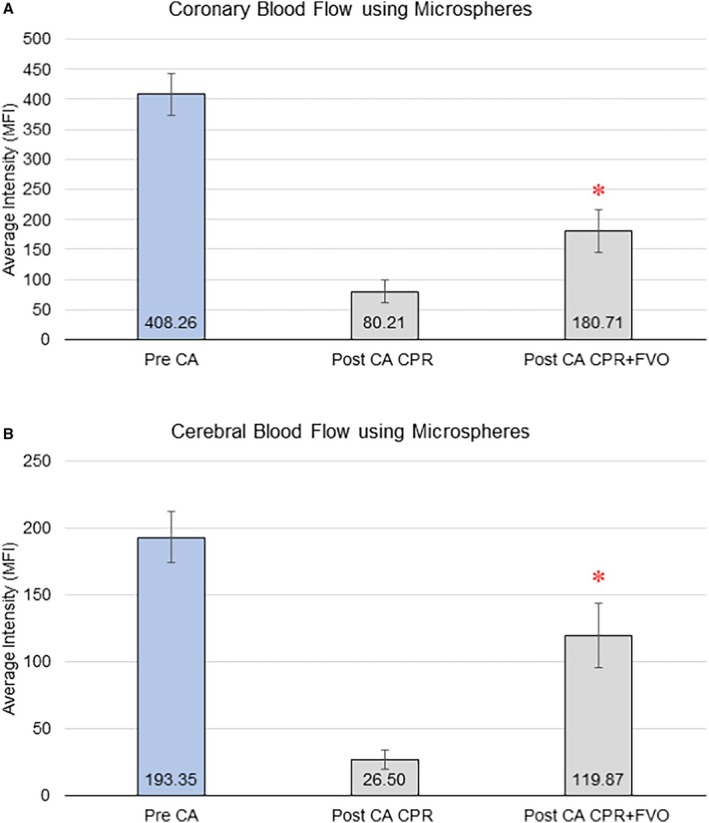
Coronary and cerebral blood flow using microspheres. MFI of (**A**) heart and (**B**) brain tissues in the CPR‐alone (n=7) vs CPR+FVO (n=6) groups, compared with pre‐CA values (n=13). The *t* test was used to compare the post‐CA CPR and post‐CA CPR+FVO values (*P<0.05). CA indicates cardiac arrest; CPR, cardiopulmonary resuscitation; FVO, femoral vessel occlusion; and MFI, mean fluorescence intensity.

**Figure 4:**
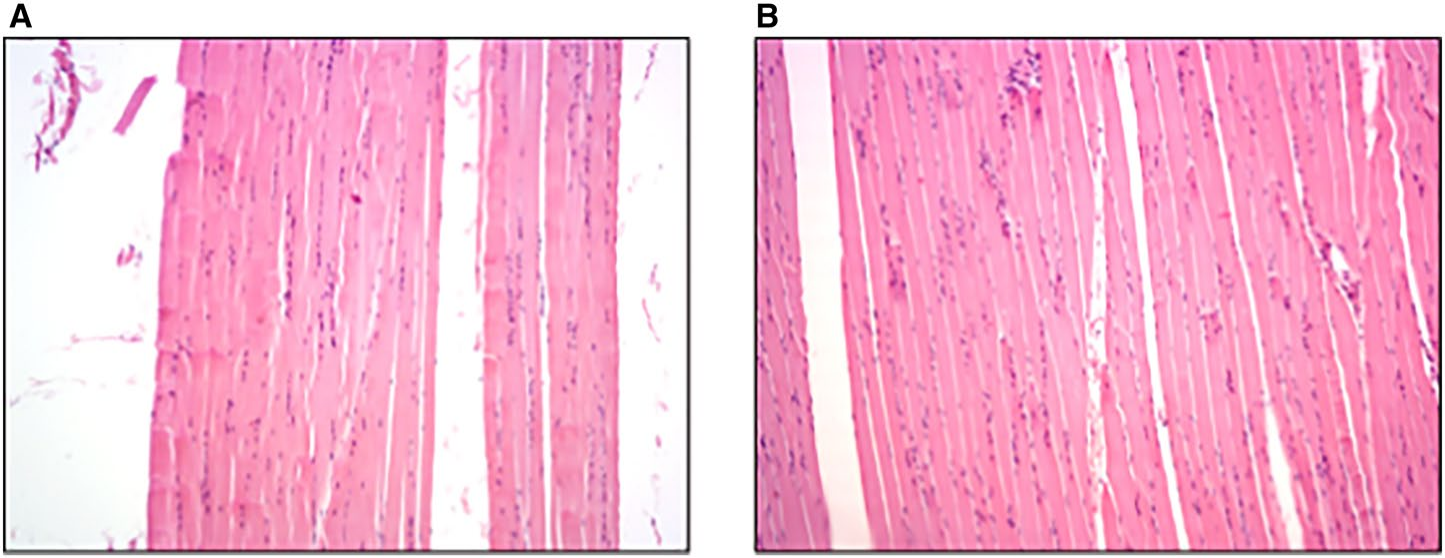
H&E stained sartorius muscle. H&E stained sartorius muscle from (**A**) CPR alone and (**B**) CPR+FVO pigs. Microscopic image in the ×10 objective lens. Slides show erythrocytes and myocyte nuclei but demonstrate no discernible pathologic differences between the control (CPR only) and experimental (CPR+FVO) groups. CPR indicates cardiopulmonary resuscitation; FVO, femoral vessel occlusion; and H&E, hematoxylin and eosin.

Perfusion in the coronary and cerebral vascular beds was quantified as the MFI measured by spectrofluorimetric analysis of microspheres embedded in the tissue samples from the animals' hearts and brains. MFI values were calculated for all animals pre‐CA and then at 5 minutes after resuscitative efforts either with CPR alone or CPR+FVO. As expected, there was a significant drop in perfusion after CA (Figure 3). During resuscitation, the experimental condition, CPR+FVO, demonstrated a statistically significant increase in MAP (Figure 2) and both myocardial MFI (*P*=0.014) and cerebral MFI (*P*=0.001) (Figure 3). Sartorius muscle tissue samples, distal to the femoral occlusion, were histologically examined by a veterinary pathologist who was blinded to the intervention, and found there were no histological differences on muscle damage, inflammation, or edema formation in samples from control and experimental animals. Representative microscopic images are presented in Figure 4.

## DISCUSSION

During CA, the lack of delivery of oxygen and essential nutrients, particularly to the brain and heart, results in high morbidity and mortality. Despite CPR, out‐of‐hospital cardiac arrest outcomes result in a fatality of 90% or more,^23^ leaving considerable room for improvement to the current standard of CA care.

Studying CA in a large mammal model is a clinically translatable way to evaluate its effects on the circulatory system and, in some instances, the central nervous system.^24^ In this study, we focused on the immediate initial resuscitation period (30 minutes), because this period of time is critically important to survival and neurologic recovery. Inspired by work using distal to proximal compression to support blood pressure,^25^ we hypothesized that focal external occlusion pressure may elicit similar effects. Focal pressure is desired for rapidity and ease of use in these emergency clinical scenarios. This study demonstrated that external FVO significantly increases MAP during native heart function and during CA, over the support of CPR alone. Crucially, there were significant improvements in cardiac and cerebral perfusion, which provides hope the FVO supplementation during CPR could decrease mortality and neurologic disability.

End‐tidal CO_2_ was also significantly increased with CPR+FVO. End‐tidal CO_2_ during CPR is a noninvasive estimate of cardiac output generated by compressions and is used clinically to measure the quality of CPR during CA.^6^ The improved end‐tidal CO_2_ indicates increased perfusion of the lungs and, thereby, enhanced gas exchange. Taken together, CPR+FVO outperforms CPR alone, and merits further exploration to augment current practices.^26^

Higher MAP has been shown to decrease neurologic morbidity following CA.^17^, ^27^ Skare et al examined the difference between a MAP of 60 mm Hg versus 90 mm Hg following return of spontaneous circulation (ROSC) on both cardiac and cerebral ischemia. They found that a MAP of 90 mm Hg translated to improved cerebral blood flow, as well as metabolic markers such as lower lactate, glucose, and pyruvate, which served as a proxy of neurologic function.^17^ With a higher MAP, increased blood flow through the cerebrum permits increased glucose metabolism and the clearance of the metabolic intermediates of glucose metabolism. Russo et al used a cerebral performance category score to measure neurological recovery following out‐of‐hospital cardiac arrest, and found that a MAP of ≥75 mm Hg was significantly associated with improved scores on their measure.^26^ Upon achieving ROSC, the American Heart Association highly recommends that patients maintain a MAP of >60 to 65 mm Hg or a systolic blood pressure >90 mm Hg to maintain adequate forward perfusion to organ tissue.

Coronary perfusion pressure (CPP), a measure of blood flow to the myocardium, is significantly correlated with CPR outcomes.^28^, ^29^ Freiss et al found that CPR directed toward maintaining CPP >20 mm Hg had a stronger association with 45‐minute intensive care unit survival after ROSC than CPR directed to achieving a specific depth (31 mm or 51 mm).^28^ Halperin et al measured myocardial recovery via echocardiography‐measured left ventricular ejection fraction following ROSC in a porcine model, and found that pigs with a higher CPP (≥30 mm Hg) had better myocardial recovery than those whose CPP was ≈12 mm Hg. Increasing coronary perfusion and myocardial blood flow has also been examined in human CPR outcomes. Paradis et al demonstrated that in human cardiac arrest cases, a CPP >15 mm Hg was most predictive of achieving ROSC and reported no survivors with a CPP <15 mm Hg in their study.^13^ In the present study, CPR+FVO supplied a statistically significant increase in MAP and coronary and cardiac perfusion. Taken together, these findings suggest a substantial improvement in CPP, and therefore improved rates of ROSC may be expected with FVO during CPR.

The increased perfusion to the coronary and cerebral vascular beds observed in the CPR+FVO pigs may be due to the minimization of perfusion of hindlimb muscle in the bilateral lower extremities, acting like an augmented vasoconstrictive response. When the body senses poor perfusion, vasoactive peptides are released to shunt blood flow to the crucial organs for survival and away from tissues more tolerant of ischemic conditions. This gives a relative increase in blood perfusion to the vital organs. One past study noted that the application of external tourniquets applied bilaterally to the lower extremities was able to return an average of 700 to 800 mL of blood to the remaining circulatory circuit during surgical cases.^30^ Our results also demonstrated a greater increase in MAP for external FVO compared with surgical FVO. We hypothesize the use of external FVO has the additional benefit of compressing collateral vessels in the proximal leg, not only the femoral artery and vein, thereby contributing to the increase in MAP. FVO‐induced supplementation of perfusion to critical organs like the brain and heart may prove to be the difference between recovery with good neurological function versus death or permanent disability.

A common concern with vascular occlusion is ischemic injury.^31^ Despite the short time interval of CA resuscitation efforts and the knowledge that during CA the lower extremities are suffering from ischemia regardless, this study sought to evaluate if additional injury was noted in the CPR+FVO group versus controls. No histological differences in muscle damage, inflammation, or edema of the sartorius muscle between the groups were detected. This finding is consistent with the greater tourniquet safety literature and the common clinical practice of tourniquet use for short‐time intervals (<2 hours). Specifically, a study evaluating prehospital extremity tourniquet use for major trauma found no difference in ischemic injury between the tourniquet group and nontourniquet group.^32^ Hanberg et al evaluated ischemic metabolite levels of tissues after tourniquet use, and found that a tourniquet duration of 1 hour had limited tissue ischemia and instant reperfusion of tissues.^33^ Notably, the use of tourniquets is common practice in orthopedic surgeries and can be inflated on the lower limbs for up to 2 hours without neurovascular injury.^34^ The duration of CPR for an acceptable neurologic outcome is <1 hour,^33^ with clinical literature, save case reports, stating optimal neurologic outcomes are found within a window of <21 to 25 minutes of CPR.^35^, ^36^

Limitations of this study include the anatomic variability present in the animals; however, this variability may better allow extrapolation to represent a larger population. There was notable hemodynamic variability between animals, which may have been overcome with additional animals. We attempted to minimize potential variability in the amount of pressure manually applied by research staff for the external occlusion by training and supervision, and designating consistent roles among the research staff. This study used a delay to the start of chest compressions after unsupported CA of 5 minutes; a prior study has used a slightly longer duration of 8 minutes of untreated cardiac arrest.^24^ Our metric for cardiac and cerebral perfusion, tissue MFI, was calculated based on microsphere injection after 5 minutes of resuscitation efforts and compared with microsphere injection during native heart function. Further research may examine additional microsphere injection time points throughout the resuscitation period to explore if persistent, greater, or diminished differences exist. Within the limitation that animals were not resuscitated and survived to examine survival or neurologic impairment, MAP was used as the primary end point, because it is a crucial indicator of perfusion pressure and is used clinically and in resuscitative research studies.^5^, ^9^, ^10^, ^11^, ^12^, ^14^, ^15^, ^17^, ^19^, ^22^, ^25^, ^27^, ^29^, ^30^, ^31^, ^32^ Although coronary perfusion pressure may be ideal, the continuous monitoring of blood pressure during resuscitation has been recommended by the American Heart Association as a CPR‐quality metric and shown to be predictive of ROSC.^37^, ^38^ Reperfusion injuries or neurologic outcomes were not examined here, because this study was designed as a terminal experiment; no animals were kept alive. The sartorius muscle biopsies were taken immediately at the conclusion of the study, and this may not have allowed adequate time to conduct a quantitative study for ischemic reperfusion injury to the tissue distal to occlusion. Furthermore, some elements of ischemic preconditioning from the phase 1 of the trial may have been present. This study focused on perfusion during resuscitation and not survival; next steps include examining if the increased perfusion demonstrated with CPR+FVO leads to ROSC or improves neurologic outcomes in animals. Future studies would also need to be conducted to measure specific changes to hindlimb perfusion and to validate translatability between a porcine model and human subjects due to lower extremity anatomical differences. Human subjects have much larger leg blood volumes compared with swine, suggesting that a clinical trial may produce effects even greater than those observed in this study.

## CONCLUSIONS

Bilateral FVO during CPR significantly increased MAP and perfusion to the heart and brain in a porcine model of CA. Because improved perfusion can decrease the mortality and morbidity of CA events, these results support FVO techniques as an adjunctive therapy and encourage further explorations to augment the success of CPR.

## Sources of Funding

This work was supported in part by the Medical University of South Carolina Idea Technology Development Grant and the South Carolina Research Authority Academic Startup Grant. H.H. was supported in part by TL1 funding: National Institutes of Health‐National Center for Advancing Translational Sciences TL1 TR00145‐08 and UL1TR001450‐08.

## Disclosures

J.K., H.H., and K.M.Q. disclose coinventor status on patent application number 206085–0104‐00US. J.K., M.L.D., H.H., and K.M.Q. disclose equity in Heartbeat Tech Corporation. All other authors and collaborators have nothing to disclose.

## Supporting information

Tables S1–S4
Figures S1–S2
