# Supplementary material for: Femoral Vessel Occlusion Enhances Cardiac and Cerebral Perfusion in a Porcine Model of Cardiac Arrest
Source: J Am Heart Assoc. 2025 Jun 23;14(13):e037413. doi: 10.1161/JAHA.124.037413 (PMC12450009; doi:10.1161/JAHA.124.037413)
Supplement: Supplementary file 1 — Tables S1–S4 Figures S1–S2 [file JAH3-14-e037413-s001.pdf]

SUPPLEMENTAL MATERIAL

TABLE S1. Arterial blood gas measurements during native heart function (pre-CA) and after five minutes of cardiac arrest (CA) with resuscitation with CPR versus CPR+FVO.

	Pre CA (n=9)	CPR (n=5)	CPR + FVO (n=4)	ANOVA p-value
pH	7.52±0.17	7.35±0.32	7.32±0.19	0.405
pCO_2, mmHg	40.69±9.35	44.88±34.36	46.68±24.79	0.932
pO_2, mmHg	482.11±109.90	217.20±140.27	173.25±250.84	0.023*
BEecf, mmol/L	9.44±6.65	-3.20±10.87	-4.50±11.64	0.052
HCO_3, mmol/L	32.49±4.31	22.06±8.05	21.73±10.67	0.040*
TCO_2, mmol/L	33.67±4.24	23.40±8.68	23.25±10.87	0.049*
SO_2, %	100.00±0.00	89.60±22.14	73.75±33.00	0.321
Na, mEq	136.78±3.73	142.60±6.43	144.50±7.53	0.098
K, mEq	3.79±0.40	3.80±1.07	3.80±1.00	0.590
iCa, mmol/L	1.34±0.11	1.16±0.31	1.04±0.31	0.215
Glu, mg/dL	81.78±25.06	83.60±26.92	92.50±50.91	0.976
Hct, %	22.56±3.84	21.00±5.36	21.67±7.14	0.592
Hb, g/dL	7.68±1.30	5.85±4.08	7.37±2.55	0.456

Values are presented as mean ± standard deviation (SD). *P-value <0.05 considered significant.

pH, logarithm of hydrogen concentration; pCO_2, partial pressure of carbon dioxide; pO_2, partial pressure of O_2; BEecf, extracellular base excess; HCO_3, Bicarbonate; TCO_2, total carbon dioxide content; SO_2%, oxygen saturation; Na, sodium; K, potassium; iCa, ionized calcium; Glu, glucose; Hct, hematocrit; Hb, hemoglobin.

TABLE S2. Arterial blood gas measurements comparing pre-CA, CPR, and CPR+FVO with Kruskal-Wallis test and three independent Mann-Whitney U tests.

		Post-CA				Mean Separation by Mann-Whitney U (p-values)		
	Pre CA (n=9)	CPR (n=5)	CPR+FVO (n=4)	ANOVA (p-value)	Kruskal-Wallis	Pre CA vs. CPR	Pre CA vs. CPR+FVO	CPR vs. CPR+FVO
pH	7.52±0.17	7.35±0.32	7.32±0.19	0.405	0.661	0.738	0.730	0.42
pCO_2, mmHg	40.69±9.35	44.88±34.36	46.68±24.79	0.932	0.825	0.606	0.736	0.905
pO_2, mmHg	482.11±109.90	217.20±140.27	173.25±250.84	0.023*	0.042*	0.007*	0.266	0.73
BEecf, mmol/L	9.44±6.65	-3.20±10.87	-4.50±11.64	0.052	0.046*	0.014*	0.159	0.905
HCO_3, mmol/L	32.49±4.31	22.06±8.05	21.73±10.67	0.04*	0.032*	0.006*	0.187	0.73
TCO_2, mmol/L	33.67±4.24	23.40±8.68	23.25±10.87	0.049*	0.042*	0.013*	0.190	0.556
SO_2, %	100.00±0.00	89.60±22.14	73.75±33.00	0.321	0.059	0.055	<0.001*	0.492
Na, mEq	136.78±3.73	142.60±6.43	144.50±7.53	0.098	0.055	0.035*	0.084	0.857
K, mEq	3.79±0.40	3.80±1.07	3.80±1.00	0.59	0.515	0.250	0.643	0.984
iCa, mmol/L	1.34±0.11	1.16±0.31	1.04±0.31	0.215	0.279	0.249	0.190	0.905
Glu, mg/dL	81.78±25.06	83.60±26.92	92.50±50.91	0.976	0.872	0.797	0.674	0.905
Hct, %	22.56±3.84	21.00±5.36	21.67±7.14	0.592	0.525	0.278	0.615	0.905
Hb, g/dL	7.68±1.30	5.85±4.08	7.37±2.55	0.456	0.828	0.601	0.636	0.857

Summary values are presented as mean ± standard deviation (SD). *P-value <0.05 considered significant.

pH, logarithm of hydrogen concentration; pCO_2, partial pressure of carbon dioxide; pO_2, partial pressure of O_2; BEecf, extracellular base excess; HCO_3, Bicarbonate; TCO_2, total carbon dioxide content; SO_2%, oxygen saturation; Na, sodium; K, potassium; iCa, ionized calcium; Glu, glucose; Hct, hematocrit; Hb, hemoglobin.

TABLE S3. Minute-by-minute change in mean arterial pressures (Delta MAP) averaged over each minute during cardiopulmonary resuscitation (CPR) alone or during CPR with concomitant femoral vein occlusion (CPR+FVO).

Minute	Average Delta MAP, mmHg CPR (n = 7)	CPR+FVO (n = 6)	
0	-26.1	-36.0	
1	-19.3	-30.4	
2	-17.5	-22.6	
3	-18.2	-27.9	
4	-19.9	-21.7	
5	-19.2	-14.5	
6	-18.7	-23.5	
7	-18.5	-23.8	
8	-21.4	-16.0	
9	-14.5	-20.8	
10	-19.3	-20.5	
11	-25.5	-20.8	
12	-19.5	-9.3	
13	-20.0	-18.2	
14	-23.9	-13.1	p = 0.0201
15	-30.2	-19.7	
16	-27.2	-17.2	
17	-27.9	-16.2	
18	-27.3	-17.9	
19	-25.1	-14.9	
20	-31.3	-11.7	
21	-27.9	-9.9	
22	-26.3	-10.2	
23	-26.5	-7.9	
24	-18.5	-5.5	
25	-20.8	-16.4	
26	-20.4	-25.3	
27	-18.9	-24.5	
28	-23.0	-18.5	
29	-23.8	-20.2	

Delta MAP is defined as the average baseline pre-cardiac arrest MAP minus the MAP at that timepoint. Cardiac arrest represents timepoint 0. Values above are the mean for all animals in each group. The P value is for the test between CPR+FVO and CPR-alone pigs during the entire 30 minutes of CPR.

Table S4. Arterial blood gas measurements during native heart function (pre-CA) and after five minutes of cardiac arrest (CA). Values are presented as means and standard deviation. P values were calculated using an ANOVA to compare all values.

	Pre CA (n=9)	CPR (n=5)	CPR + FVO (n=4)	ANOVA p-values
pH	7.52±0.17	7.35±0.32	7.32±0.19	0.405
pCO_2, mmHg	40.69±9.35	44.88±34.36	46.68±24.79	0.932
pO_2, mmHg	482.11±109.90	217.20±140.27	173.25±250.84	0.023*
BEecf, mmol/L	9.44±6.65	-3.20±10.87	-4.50±11.64	0.052
HCO_3, mmol/L	32.49±4.31	22.06±8.05	21.73±10.67	0.040*
TCO_2, mmol/L	33.67±4.24	23.40±8.68	23.25±10.87	0.049*
SO_2, %	100.00±0.00	89.60±22.14	73.75±33.00	0.321
Na, mEq	136.78±3.73	142.60±6.43	144.50±7.53	0.098
K, mEq	3.79±0.40	3.80±1.07	3.80±1.00	0.590
iCa, mmol/L	1.34±0.11	1.16±0.31	1.04±0.31	0.215
Glu, mg/dL	81.78±25.06	83.60±26.92	92.50±50.91	0.976
Hct, %	22.56±3.84	21.00±5.36	21.67±7.14	0.592
Hb, g/dL	7.68±1.30	5.85±4.08	7.37±2.55	0.456

Values are presented as mean ± standard deviation (SD). *P-value <0.05 considered significant.

pH, logarithm of hydrogen concentration; pCO_2, partial pressure of carbon dioxide; pO_2, partial pressure of O_2; BEecf, extracellular base excess; HCO_3, Bicarbonate; TCO_2, total carbon dioxide content; SO_2%, oxygen saturation; Na, sodium; K, potassium; iCa, ionized calcium; Glu, glucose; Hct, hematocrit; Hb, hemoglobin.

Figure S1. Power analysis to determine sample size.

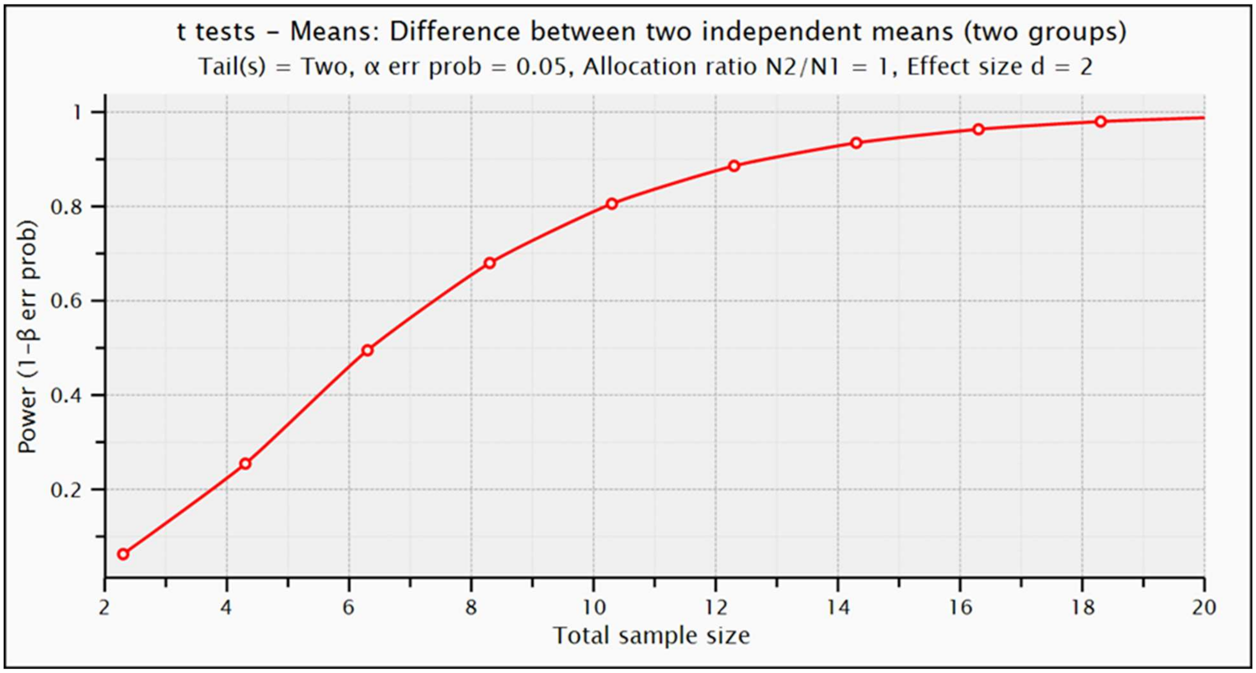

A pooled standard deviation of 10mmHg was used, with alpha (risk of Type I error) set at 0.05 and power (1-beta) set at 0.8.).

Figure S2. Delta Mean Arterial Pressure during Cardiac Arrest.

A

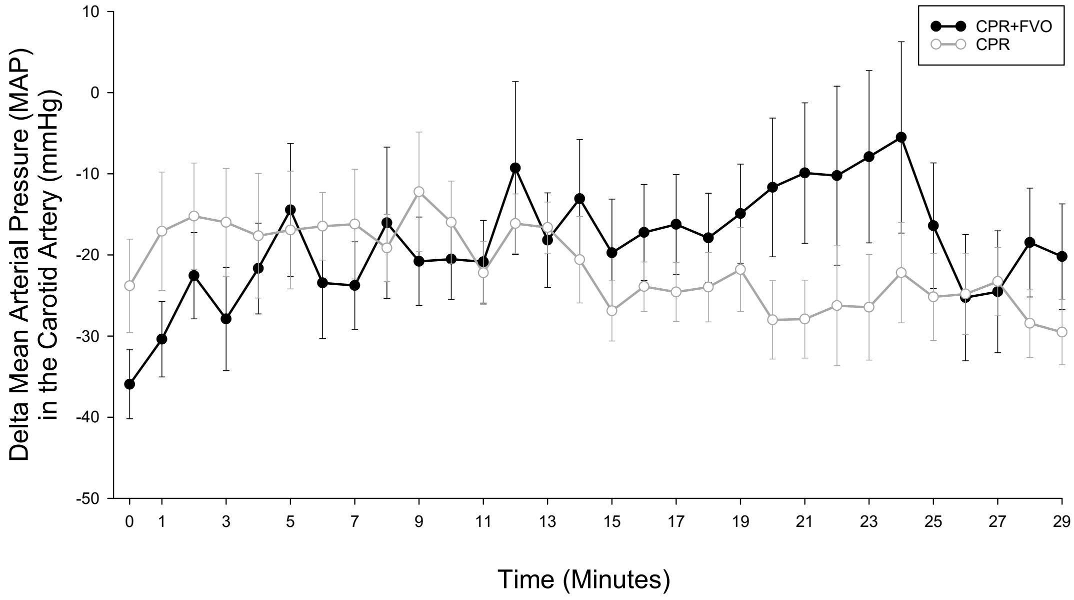

B

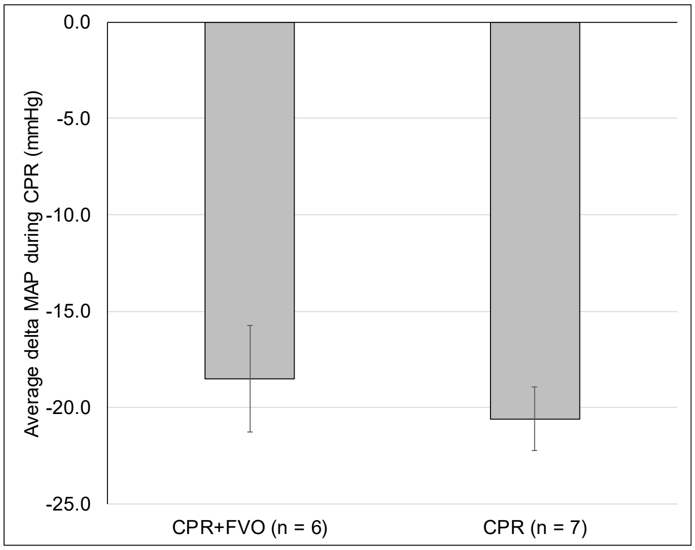

(A) Delta MAP measured in the carotid artery during cardiac arrest (average baseline pre-cardiac arrest MAP - measured minute MAP during cardiac arrest) for each pig in the CPR only group (n=6) and CPR+FVO group (n=7). (**B)** Means (bars) and standard deviation (error bars) for the delta MAP during the CPR period in the CPR only and CPR+FVO groups (t-test).
